# Complete genome sequence of *Limnothrix* sp. CMB-01, a self-flocculating cyanobacterium isolated from rare earth element wastewater

**DOI:** 10.1128/mra.00185-26

**Published:** 2026-03-23

**Authors:** Shanren Li, Dandan Che, Ming Li, Zhefu Li, Xibin Qiu, Mingzi Wang

**Affiliations:** 1 National and Local United Engineering Research Center of Industrial Microbiology and Fermentation Technology, College of Life Sciences, Fujian Normal University 12425, Fuzhou, China; 2 Appraisal Center for Environment and Engineering, Ministry of Ecology and Environment of China 90403 , Beijing, China; Queens College Department of Biology, Queens, New York, USA

**Keywords:** *Limnothrix*, complete genome, self-flocculating cyanobacterium

## Abstract

We report the complete genome sequence of *Limnothrix* sp. CMB-01, a self-flocculating cyanobacterium isolated from rare earth element wastewater. The assembled genome comprises a single circular chromosome of 4,574,928 bp with a GC content of 55.22%, providing genomic insights into the metabolic potential and adaptive traits of this underexplored cyanobacterium.

## ANNOUNCEMENT

Wastewater generated from rare earth element (REE) production exhibits high ammonia nitrogen concentrations, underscoring the urgent need for efficient and sustainable treatment strategies. Self-flocculating microalgae offer a promising bioremediation approach, enabling effective ammonia nitrogen removal and low-energy harvesting through natural aggregation and sedimentation (1–3). Although cyanobacteria of the genus *Limnothrix* are widely distributed in aquatic environments, genomic resources for strains with bioremediation potential remain limited. Strain CMB-01 was isolated from REE production wastewater collected in Changting County, Longyan City, Fujian Province, China (25.74°N, 116.36°E). Isolation was performed by serially diluting the wastewater sample with sterile water, plating onto BG11 agar plates, and incubating at 25 °C for 10 days under continuous illumination (75 μmol m⁻² s⁻¹). A single colony was subsequently purified on BG11 plates.

Genomic DNA of strain CMB-01 was sequenced using a hybrid approach combining Oxford Nanopore (long-read) and DNBSEQ (short-read) platforms at Sangon Biotech (Shanghai, China). The strain was cultured in BG11 medium under continuous illumination (75 μmol m⁻² s⁻¹) with shaking at 110 rpm and 25°C. DNA was extracted using the NucleoBond HMW DNA kit (Macherey-Nagel, Germany) and quantified using a NanoDrop One spectrophotometer and Qubit 4.0 fluorometer (Thermo Fisher Scientific, USA). DNA integrity and purity were assessed by 0.75% agarose gel electrophoresis.

For long-read sequencing, a DNA library was prepared with the SQK-LSK110 ligation sequencing kit without DNA shearing and sequenced on a PromethION R9.4.1 flow cell. Basecalling, barcode segmentation, and adapter trimming were performed using Guppy v6.5.6 (4) in high-accuracy mode, generating 95,402 raw reads (average length = 10,551 bp, *N*_50_ = 21,764 bp). Raw reads were filtered using Filtlong v0.2.1 (5) to remove short (<1,000 bp) and low-quality reads (*Q* < 6), resulting in 88,009 subreads (average length = 11,294 bp; *N*_50_ = 21,831 bp). For short-read sequencing, DNA was sheared to approximately 300 bp, and a 150 bp paired-end library was prepared using the MGI Easy DNA Library Prep Kit and sequenced on a DNBSEQ-T7 instrument, generating 39,408,506 raw reads (5.91 Gb total). Raw reads were processed using fastp v0.23.0 (6) for adapter trimming and quality filtering, resulting in 39,404,298 clean reads (5.88 Gb total).

Filtered long reads were assembled using Canu v2.2 (7), and the resulting contig was circularized with Circlator v1.5.5 (8). Gap filling and sequence correction were performed by integrating short reads using GapFiller v1.11 (9) and PrInSeS-G v1.0.0 (10), respectively. The assembly was iteratively polished with Pilon v1.22 (11) until no further errors were detected. CheckM v1.2.4 (12) estimated genome completeness at 98.28% with 0.16% contamination, and average genome coverage was approximately 894.73×.

The final assembly yielded a single circular chromosome of 4,574,928 bp with a G + C content of 55.22% (unrotated). Gene annotation was performed using the NCBI Prokaryotic Genome Annotation Pipeline v6.10 (13). The 16S rRNA gene sequence showed 99.00% identity to known *Limnothrix* species, suggesting that strain CMB-01 represents a novel strain within the genus. Default parameters were used for all software unless otherwise noted. The principal genomic features are summarized in Table 1.

**TABLE 1:** Genome assembly and annotation statistics for *Limnothrix* sp. CMB-01

Parameter	Value
GenBank accession number	CM143720
Genome size (bp)	4,574,928
GC content (%)	55.22
*N*_50_ (bp)	4,574,928
Coverage (×)	894.73
Genes (total)	3,789
CDSs (total)	3,735
CDSs (with protein)	3,668
CDSs (without protein)	67
rRNA genes (5S, 16S, 23S)	6 (2, 2, 2)
tRNA genes	44
Noncoding RNAs	4
Pseudogenes	68

## Data Availability

The complete genome sequence of *Limnothrix* sp. CMB-01 has been deposited in NCBI GenBank under accession number CM143720 (BioProject number PRJNA1420451 and BioSample number SAMN55230565). The raw sequence data are available in the Sequence Read Archive under accession numbers SRR37182836 (ONT) and SRR37182837 (DNBSEQ), respectively.
